# Fibrosis following Acute Skeletal Muscle Injury: Mitigation and Reversal Potential in the Clinic

**DOI:** 10.1155/2020/7059057

**Published:** 2020-09-01

**Authors:** Tyler Gardner, Keith Kenter, Yong Li

**Affiliations:** ^1^Department of Orthopaedic Surgery and Sports Medicine at the University of Cincinnati, Cincinnati, OH, USA; ^2^Department of Orthopaedic Surgery at Western Michigan University Homer Stryker MD School of Medicine, Kalamazoo, MI, USA

## Abstract

Skeletal muscle injuries occur often in athletics and in daily life. In minor injuries, muscles are able to regenerate completely and recover their functional capabilities. However, in the case of severe injuries, the injured muscle cannot recover to a functional level because of the formation of fibrous scar tissue. The physical barrier of scars is significantly challenged in both research and clinical treatment. Fibrous scar tissue not only limits cells' migration, but also contributes to normal tissue biomechanical properties. This scar formation creates an unsuitable environment for tissue structure resulting in frequent pain. Antifibrosis treatment is one of the major strategies used to augment muscle regeneration and accelerate its functional recovery. This review will discuss the currently available methods for improving muscle regeneration with a specific focus on antifibrosis applications. We also discussed several novel hypotheses and clinical applications in muscle fibrosis treatment currently in practice.

## 1. Introduction

Skeletal muscle injuries are common injuries experienced by athletes of all levels. Muscle strain is extremely common and usually occurs due to eccentric contractions and overstraining during activity [1, 2]. Particularly, sports that involve springing or jumping are commonly implicated in muscle strain injuries. Skeletal muscle does have the capability to heal itself; however, the process of healing can be incomplete and lead to a decrease in function and risk of repeat injury. Skeletal muscle injuries also often occur in the aged populations, leading to inconvenience in daily life. The slow healing of aged muscle is caused by both losing muscle mass, fibrosis, and systemic age [3, 4].

Skeletal muscle is one of the largest tissues by mass in the human body, making up 40–45% of total body weight. The primary function is the production of motion and support of the bony skeleton. In order to do so, skeletal muscles are made up of multiple basic structures. Myofibers are the basic component within muscles. It contains the various of muscle cells (e.g., myoblasts and progenitor cells) and fiber typical cytoplasm and organelles [5]. Myofibers are formed when multiple muscle progenitor cells (muscle satellite cells or muscle stem cells—MuSCs) fuse to form myotubes. In this process, they resemble long cylinders. The myotubes mature to form myofibers, which is noticeable when the nuclei move from its central location to a peripheral, subsarcolemmal location. Myofibers contain multiple nuclei due to the syncytial nature.

Multiple layers of connective tissue are associated with skeletal muscle. The layer surrounding individual myofibers is termed the endomysium. Bundles of individual myofibers are bundled into fascicles, which are surrounded by perimysium. Surrounding individual muscles is the epimysium. The endomysium is made up of type I and III collagen and connects to the basement membrane of the myofibers of which is composed of type IV and VI collagen [6]. The endomysium transmits forces to tendon and surrounding muscle fibers [7]. In individual muscles, fiber arrangement determines function and contractile properties and can be parallel or oblique [1, 7]. The functional compartment of limb muscle also contains a network support, such as vascular trees and neuron muscle junctions (NMJs).

## 2. Muscle Injury and Repair

**Figure 1:**
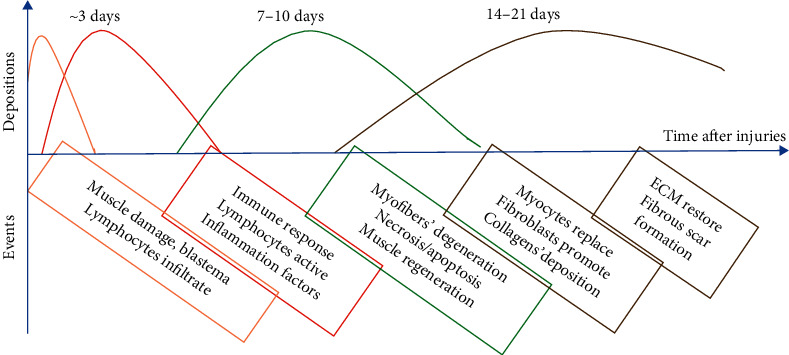
The time relation to muscle response after injuries.

Muscle injuries were estimated to cost greater than $790B based on the published data in 2014 [8]. Muscle may be injured by either direct trauma or from physiologic sequelae. Direct trauma includes lacerations, contusions, or strains. Indirect trauma is due to ischemia or neurological dysfunction or systemic diseases. Once muscle injury occurs, a relatively orderly process occurs (Figure 1) [1, 2]. If due to mechanical trauma, the integrity of the myofiber plasma membrane is disrupted. This leads to the movement of extracellular calcium intracellularly and autodigestion by intrinsic proteases. Local swelling and hematoma formation promote degeneration as well and occur soon after the event. Inflammation leads to inflammatory cell movement into the area of concern and includes macrophages, T-cells, and neutrophils [9, 10]. A variety of cytokines are secreted by these cells, which further propagates the inflammatory event.

The healing process occurs in stages. In the first few days following injury, active muscle degeneration and inflammation occur. Neutrophils enter the site due to cytokines released from damaged tissue [9, 10]. The neutrophils phagocytose injured cells and enhance the inflammatory response. Monocytes and macrophages are attracted by cytokines released by neutrophils. The cytokines associated with this phase are Tumor Necrosis Factor- (TNF-) alpha and Interleukin- (IL-) 6. IL-6 and Insulin-Like Growth Factor- (IGF-) 1 and are associated with satellite cell differentiation and proliferation [11].

Muscle regeneration usually starts 7–10 days after the injury, with the process peaking at 2 weeks and slowing or decreasing at weeks 3-4. Monocytes and macrophages, attracted to the injury site as described above, propagate the process of healing and regeneration. Macrophages have 2 phenotypes. The M1 phenotype is proinflammatory, and the M2 phenotype is profibrotic. Proper muscle healing requires a balance of the two phenotypes [12, 13]. In the early stages, the M1 macrophage response is driven by T-helper 1 cells. This initial inflammation is important for the proliferation of progenitor cells [14]. However, unregulated M1 response can lead to muscle damage, which has been linked with nitric oxide release [15].

Fibrosis happens when a scar tissue begins to form between weeks 2 and 3 after injury and increases or decreases with timing. Transforming growth factors (TGFs) are considered major stimulator during fibrosis formation [16, 17]. Once fibrosis occurs, a complete regeneration of muscle function is affected negatively. M2 macrophages are propagated by T-helper 2 cells. These cells, while being important for healing and controlling inflammation from M1 macrophages, can also induce fibrosis [18, 19]. TGF-*β*1 is a cytokine released from M2 macrophages and has been implicated in fibrosis as a key factor. TGF-*β*1 activates fibroblasts, which then leads to pathologic deposition of collagen into the extracellular matrix (ECM) [17]. Although extracellular components are important to tissue integrity, excessive deposition leads to poor recovery of function.

## 3. Clinically Mitigating Fibrosis Acutely

Movement is important regarding mitigation of fibrosis. Some animal models have shown that passive movements have led to a reduction of fibrosis in healing tendons [20]. Other models looking at active stretching exercises showed reduced subcutaneous collagen formation after injury [21, 22]. Using an ex vivo model, it was shown that a brief tissue stretch decreased soluble TGF-*β*1 [23]. The same study, in an in vivo model using type-1 procollagen as a measurement of fibrosis, showed that there was an increase in procollagen in the absence of stretch. Although more models are needed, controlled stretching programs could translate to a good treatment modality for the prevention and treatment of fibrosis. Similar studies have been developed in rehabilitation and physical therapies.

NSAIDs are a common class of medications used acutely for treatment of pain and inflammation in skeletal muscle injuries. These medications inhibit cyclooxygenases (COX), which have 3 isoforms. COX-1 is constitutively active and produces prostaglandins from arachidonic acid. COX-2 produces prostaglandins as well, but is induced [3]. Although these medications are successful in the treatment of pain, inhibition can lead to negative consequences for long-term healing [10]. Some reports have demonstrated that nonsteroidal anti-inflammatory drugs (NSAIDs) can delay and impair skeletal muscle recovery [9, 10]. Inhibition of COX-1 and COX-2, either by themselves or together, can lead to reduced differentiation and fusion of satellite cells [9]. Given that inhibition of COX-2 alone still causes this effect, even use of selective COX-2 inhibitors could lead to negative effects on skeletal muscle healing [24].

Cyclooxygenases are not the only enzymes in the arachidonic acid pathway. When COX enzymes are blocked, substrates are shunted to lipoxygenases. This pathway creates molecules called leukotrienes [25]. Leukotrienes are similar to prostaglandins, as they cause inflammation and produce an immune response. One study used a medication, licofelone, which inhibits lipoxygenase (5-LOX) and COX enzymes [26]. In this study, licofelone reduced functional muscle degeneration and enhanced tendon healing. This further proves that leukotrienes could be the culprit of the negative effects of NSAIDs on muscle healing and prove to be a possible treatment in the future.

Multiple mechanisms have been researched for the mitigation of fibrosis in the acute phase. Cytokines or/and growth factors that are secreted by inflammatory cells can induce and propagate fibrosis [9–11, 17–19]. Thus, the targeting of them is one of the most studied forms of treatment [10, 16, 19]. Given the complicated nature of the process, there are numerous therapeutic potentials to target the pathological fibrosis.

Platelet-rich plasma (PRP) has been studied for multiple uses, one of which is for using to prevent or treat fibrosis. To create it, the platelet concentration from the plasma fraction (usually from the patient) is concentrated to 1.5–8 times the physiological amount [27]. The platelets are best used when activated, which is when the presence of growth factors and cytokines is at its highest. There are two types of PRP based on leukocyte concentration—leukocyte-rich (LR-PRP) or leukocyte-poor (LP-PRP). Benefits of this modality are its use in autologous samples, direct injection into the tissues, and a good safety profile. However, controversial results from different study groups have been reported [28–30].

PRP can have direct effects on the microstructure of muscle. Myofibroblasts have features of both smooth muscle and fibroblasts, as they have bundles of actin and myosin, large focal adhesion complexes, and express alpha-Smooth Muscle Actin (SMA). In vitro evaluations have demonstrated that PRP limits the transition of fibroblastic cells into myofibroblastic cells. This has been preliminarily shown due to its interference with the intracellular signaling of TGF-*β*1 [27]. Unfortunately, this study did not differentiate between LR-PRP and LP-PRP. The target of therapeutics would be limiting the effect of these cells, as they are important to tissue integrity after injury but can become pathologic if not well controlled. PRP, both alone and in conjunction with bone-marrow-derived mesenchymal stromal cells, stimulates myogenic progenitor cells in skeletal muscle tissue [28]. However, the variations in the concentration and dosing of PRP itself can result in different effects on the muscle. This must be considered during the preparation of PRP. One study investigating the use of PRP in craniofacial bone repair showed an increase in myofibroblastic cells, associated with TGF-*β* present in PRP [31]. This would have obvious negative effects, as myofibroblastic cells would create a more fibrotic milieu.

## 4. Treatments Targeting Reversal of Fibrosis

One aspect of the literature that has not been explored in depth discussion is the treatment of fibrosis after it has been occurred. Multiple cytokines, proteins, and cells have been discovered as both culprits and targets for therapeutic potential.

Bone Morphogenetic Protein- (BMP-) 7 was shown in one study to prevent and/or reverse TGF-*β*1-dependent myofibroblast differentiation with a dose-dependent and time-dependent effect [32]. This effect is due to BMP-7 and its effect on endosomes. The same study showed that CD44, a cell marker, has different isoforms and can have different effects on fibrosis through modulation of BMP-7 [32]. This could have use in the future as a target for the treatment of chronic fibrosis.

Matrix metalloproteinases (MMPs) are a group of proteases, of which 24 different genes have been discovered, whose function is both the degradation of components of the ECM and the regulation of extracellular tissue signaling networks [33]. MMPs are located diffusely throughout the body and in multiple tissues. They are activated by calcium and are zinc-dependent [34]. Different MMPs have different substrates and can be broken down into subgroups based on those substrates. The different subgroups are collagenases, gelatinases, stromelysins, matrilysins, membrane-type, and other [33].

The catalytic activity of MMPs is controlled by four mechanisms. MMP activity can be controlled by either gene expression with transcriptional and posttranscriptional regulation, extracellular localization and tissue/cell release (compartmentalization), proenzyme activation, and/or inhibition by specific or nonspecific inhibitors. Specific tissue inhibitors of metalloproteinases (TIMPs) modulate the activity of MMPs [33]. MMPs have been discovered in the ability of resident muscle stem cells to migration and homeostasis [35]. They have also been implicated in systemic sclerosis, with either an increase or a decrease in levels leading to sclerosis of tissue [36].

Specifically, MMPs and TIMPs have various roles in skeletal muscle. They are important in the migration and differentiation of myoblasts [34]. Satellite cells must migrate to the injured site, which means that ECM degradation is important to this process. MMP-1 has been shown to enhance myoblast migration and differentiation [37, 38]. This is through increased expression of migration marker proteins *N*-cadherin and beta-catenin along with pre-MMP-2 and TIMP. This enhanced migration is thought to be due to elimination of ECM and cell surface components that bind, hindering fusion between two membranes [34]. MMPs have also been shown to be involved in myotube formation, specifically MMP-2, -7, -9, and MT1-MMP. MMP-2 and MT1-MMP deficiency is associated with a lack of myotube formation in culture. MMP-9 has been found to be at low levels, while TIMP-1 is abundant in basal lamina and endomysium [39, 40].

MMP-1 has also been studied specifically in skeletal muscle repair. It has been shown in a mouse model, a single treatment with MMP-1 led to enhanced muscle regeneration and decreased pathologic deposition of fibrotic components of ECM [41, 42]. The time frame of injection of MMP-1 is also important for muscle regeneration. Specifically, week 3 after injury was discovered to be the most efficacious time point, as this is when regeneration declines and fibrosis occurs. A limit to this is the fact that the study itself did not find functional results in the short testing time frame, such as an increase in peak muscle force [41, 42]. Other mouse models have shown a similar effect, with an increase in preservation of soft tissues in digit regeneration [43]. In this model, regeneration of soft tissues and wound closure rate improved. With this effect, skeletal tissue was not affected negatively. This is thought to be due to the ratio of MMPs to TIMPs in the treatment group. When studied for the treatment of Duchenne Muscular Dystrophy (DMD), diseased mice with MMP-1 gene transfer had improved myoblast transplantation efficacy [37]. Future use of this treatment or pathway could use more study.

Regarding future therapeutic targeting of MMPs, it is important to consider the biological balance required for optimal outcome. Collagen, although implicated in fibrosis, is also important for stability of repair. More importantly, ratio of MMP and TIMP expression can positively or negatively affect the stability of the tissue. In the disease of DMD, increased mRNA expression of TIMP-1 and -2 has been detected in these affected skeletal muscles [34]. Suggestions for targeting include prolonging MMP presence and using growth factors or nontoxic chemical agents to promote expression. Suggested methods include IV injection, gene therapy, or combining degradable polymers.

Fibroblasts are one of the most important cells in the fibrosis pathway, as they are pivotal to the excessive ECM protein synthesis and deposition associated with fibrosis and tissue dysfunction [44]. Fibroblasts come from either resident tissue, epithelial-to-mesenchymal transition, or circulating fibrocytes. Fibrocytes come from the circulation and are monocyte derived, with features of both monocytes and fibroblasts. Their concentration in tissue is determined by the environment and injury type [45]. In one study, it was found that the differentiation of fibrocytes occurs most likely in injured muscle [44]. T-cells are important for their differentiation. Th1-associated cytokines (INF-gamma and IL-12) inhibit fibrocyte differentiation and Th2-associated cytokines (IL-4, Il-13, and TGF-*β*1) promote fibrocyte differentiation. It also has been demonstrated that other cells (such as macrophages) can switch from proinflammatory to proregenerative later during the process of muscle injury repair [12, 46]. Overall, targeting and modulating fibrocyte function may be a possible therapeutic target to reverse muscle fibrosis after injury.

## 5. Potential Future Treatments

**Figure 2:**
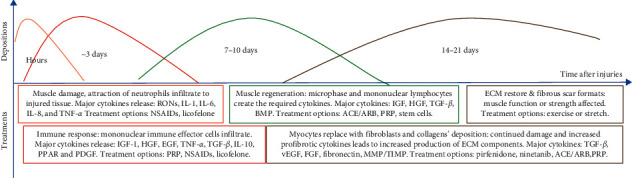
The timeline of muscle healing processes and therapeutic potential strategies.

Tissue fibrosis is not an uncommon outcome after injury and is not isolated to the muscle system. This common pathophysiologic outcome can offer potential medications for fibrosis treatment in one organ system to have crossover treatment to another organ system. However, both preclinical and clinical trials are important for the determination of efficacy and safety. Several candidate drugs have been used for clinical trials in treating muscle fibrosis (Figure 2).

Losartan (Cozaar) is an angiotensin II receptor antagonist used to treat diseases with high blood pressure or hypertension [47, 48]. Losartan administration can reduce fibrosis in several tissues, including liver, lung, kidney, and injured skeletal muscles [49, 50]. Mechanism studies suggest that Losartan is able to conjugate directly to angiotensin receptors and blocks signaling transduction, which includes Smad2/3 pathways. A clinical study also discovered that Losartan can reduce inflammation and improve immune recovery, which may indirectly prevent fibrosis in the treated tissues [51]. However, some risks of use of this medication would be changes in the blood pressure of normotensive individuals.

Pirfenidone, an antifibrotic, antioxidant, and anti-inflammatory agent, is a commonly used medication for the treatment of idiopathic pulmonary fibrosis (IPF) [52–54]. Pirfenidone has also found use in kidney, hepatic, and cardiac fibrosis. In a study of retinal pigment epithelial cells, the antifibrotic effect was attributed to the modulation of TGF-*β* signaling by preventing nuclear accumulation of Smad complexes [55]. Another study attributed this medication's effect to inhibition of the profibrotic hedgehog (Hh) signaling pathway. This inhibition is due to destabilization of the glioma-associated oncogene homolog (GLI2) protein [56]. Given the prevalence of information implicating the TGF-*β* pathway in skeletal muscle fibrosis, it is possible that pirfenidone could be used for treatment. Associated side effects with this medication are mostly gastrointestinal and skin-related, with wound healing and bleeding issues after surgery being particularly pertinent if the patient underwent an operation [57, 58]. These side effects were mitigated by dose modification, especially dose reductions and interruption.

Nintedanib is another medication used in IPF that could have possible applications regarding skeletal muscle fibrosis. This medication is a nonspecific tyrosine-kinase inhibitor with action against platelet-derived growth factor (PDGF), fibroblast growth factor (FGF), and vascular endothelial growth factor (VEGF) [54]. In a study of dermal fibroblasts, nintedanib prevented the proliferation and migration of these cells. This could have potential positive effects for skeletal muscle fibrosis, as fibroblasts, TGF-*β*, and VEGF have all be possibly implicated in the pathology [59, 60]. Side effects associated with this medication are diarrhea, bleeding issues, and liver enzyme elevation [61]. These effects were mitigated with dose reduction.

Aging increases tissue fibrosis potential globally, including the musculoskeletal system [8, 11]. Research of this process has reported that angiotensin II is associated with local fibrosis in skeletal muscles [47]. A study has indicated that the use of ACE inhibitors or ARBs can lead to decreased fibrosis and improved healing after myocardial infarction [62]. This same effect was also found with skeletal muscle injuries. Patients treated with angiotensin modulators had reduced fibrosis and improved skeletal muscle regeneration [47]. Specifically, patients with hamstring injuries were treated with Losartan (ARB), resulting in improved muscle healing without negative side effects.

## 6. Conclusion

Overall, fibrosis in muscle injury is a pathologic effect of an overly exuberant normal response. Although fibroblasts and fibrocytes are important for the strength and integrity of the healed tissue, overexpression of these cells leads to incomplete healing. Multiple mechanisms have been shown as possible targets for decreasing fibrosis. However, treating fibrosis after it has occurred is challenging. The fibrous scar tissue is important for the integrity of the healed tissue in early phases of healing; however, its presence later in the process of healing has a detrimental influence on function. Possible targets for chronic fibrosis are using cytokines to break down the excess ECM while allowing the muscle tissue to heal in place of the fibrous scar tissue. However, controlling the environment and the timing of this control are important, as these proteins are in delicate balance with one another. Fibrosis is not simply a chronic, stable condition. It is a condition that requires constant stimulus, and changing that stimulus has a possibility to reverse the pathology.
